# Ovariectomy Impairs Socio-Cognitive Functions in Dogs

**DOI:** 10.3390/ani9020058

**Published:** 2019-02-14

**Authors:** Anna Scandurra, Alessandra Alterisio, Anna Di Cosmo, Antonio D’Ambrosio, Biagio D’Aniello

**Affiliations:** 1 Department of Biology, University of Naples Federico II, Via Cinthia, 80126 Naples, Italy; annascan@hotmail.it (A.S.); alessandra.alterisio@unina.it (A.A.); dicosmo@unina.it (A.D.C.); 2 Department of Economics and Statistics, University of Naples Federico II, Via Cinthia, 80126 Naples, Italy; antonio.dambrosio@unina.it

**Keywords:** ovarian hormone, cognition, cue-following task, dog, gonadectomy, human-dog communication, pointing

## Abstract

**Simple Summary:**

The aim of this study was to test whether ovariectomy in dogs (*Canis lupus familiaris*) could impair a female’s ability in a socio-cognitive task. Forty pet dogs (18 intact females (IF) and 22 gonadectomized females (GF)) were tested in the object choice task paradigm using a human proximal pointing gesture. For the analysis, the frequency of correct, wrong and no-choices was collected; moreover, the latency of the correct choices was also considered. The IF group followed the pointing gestures more often than the GF group and with a lower latency whereas a significantly higher no-choice frequency was recorded for the GF group.

**Abstract:**

Recent studies have underlined the effect of ovariectomy on the spatial cognition of female dogs, with ovariectomized dogs showing a clear preference for an egocentric rather than an allocentric navigation strategy whereas intact females did not show preferences. Intact females had better performances than gonadectomized females in solving a learning task in a maze. Ovariectomy also affects socio-cognitive abilities, reducing the dog’s level of attention on the owner. We tested dogs (*Canis lupus familiaris*) in the object choice task paradigm to assess whether an ovariectomy could impair females’ ability to follow human signals. Forty pet dogs (18 intact females (IF) and 22 gonadectomized females (GF)) were tested in the object choice task paradigm using the human proximal pointing gesture. For the analysis, the frequency of correct, wrong and no-choices was collected; moreover, the latency of the correct choices was also considered. The IF group followed the pointing gestures more often than the GF group and with a lower latency, whereas a significantly higher no-choice frequency was recorded for the GF group. These results show a detrimental effect of ovariectomy on dogs’ socio-cognitive skills related to the responsiveness to human pointing gestures.

## 1. Introduction

Behavioral studies on the effect of the removal of ovarian steroids have showed detrimental outcomes on learning, working and reference memories, and object and social recognition in female rats. It has been shown that in ovariectomized rats, sex steroid administration reverses these effects. On the other side, rats in proestrus and estrus show better cognitive performance, suggesting that increased circulating female sex hormones may be beneficial (reviewed in [1]). Dogs are good models to study the effect of ovariectomy on cognitive performance because sterilization in bitches is very common and they are relatively simple to manage in behavioral experiments. Recent studies in a plus-maze paradigm underlined an effect of ovariectomy on the spatial navigation. Particularly, ovariectomized dogs showed a clear preference for an egocentric navigation strategy (i.e., based on their motor responses) rather than an allocentric strategy (i.e., based on the position of objects in space), whereas intact females did not show preferences [2]. Furthermore, intact females had better performances than gonadectomized females in solving a learning task in a T-maze paradigm [3]. Apart from spatial cognition, ovariectomy also affects socio-cognitive abilities reducing the dog’s level of attention on the owner in an unknown place, where stimuli competing with the owner were present [4]. In the latter research, the owners provided no signals, and they were unaware of the dog’s behavior. On the other hand, dogs are very sensitive to human gestures, which appear more significant when compared with other signals such as verbal commands [5,6,7]. The question we addressed in the present study was whether ovariectomy affects a dog’s ability to receive and respond to human gestures when intentionally solicited. One of the experimental paradigms for studying dogs’ ability to follow human gestures is the so-called “object choice task paradigm”, in which the location of an object or food, hidden to the dogs’ view, is indicated by a human model through different gestures (e.g., gazing, pointing). According to some authors [8], dogs successfully perform this task as an effect of the domestication process (“domestication hypothesis”) during which dogs could have acquired a set of social-cognitive abilities that enables them to communicate with humans in unique ways. However, the “two-stages hypothesis” assumes that ontogenetic experiences are crucial for responsiveness to the human pointing gesture [9]. Indeed, deprivation of human interaction impairs the dog’s skill to follow this signal [10] and reduces the tendency of dogs to relate to humans during solving a task [11]. This implies that an important learning component underpins the optimal performance in following the human pointing gesture. In the present study, we tested the hypothesis that ovariectomy could impair a female dog’s ability to comply with human signals. To this aim, intact and ovariectomized pet dogs were compared in the object choice task paradigm using the human pointing gesture. Considering the impaired cognitive skills observed in ovariectomized female dogs [3,4], a detrimental effect of gonadectomy on the response capability to human pointing gestures was expected.

## 2. Materials and Methods

### 2.1. Subjects

A total of 40 female never-pregnant pet dogs were tested: 18 intact females (IF, 15 Labrador retrievers and 3 Golden retrievers; 21.8 ± 6.6 months old, in anoestrous phase) and 22 gonadectomized females (GF, 19 Labrador retrievers and 3 Golden retrievers; 19.9 ± 5.1 months old; the average age of sterilization was 13.1 ± 2.0 months old). The eligibility requirements to be enrolled in the trial were: to be reared as puppies in human families; living stably at home (i.e., dogs spending time partially or totally in the garden were excluded); being younger than three years; being spayed after completing at least the first oestrous cycle. For our experimental sample, we chose Labradors and Golden retrievers because they are the most popular family dog breeds allowing us to obtain a decent sample size. Furthermore, they are among the most sociable, curious and bold breeds [12,13], thus eliminating any problem related to pre-selection of individuals. Volunteers were recruited from our personal database, direct contacts and via the Internet. 

### 2.2. Experimental Procedure

All dogs fasted for at least 4 h, and were tested in a room (about 14 m^2^) unknown to them, at the University of Naples Federico II. Before the test, the dogs could enter the room for approximately 5 min to freely explore and become familiar with the environment and the researchers. Three experimenters (E1, E2, E3) were involved in the trial: E1 held the dog, E2, the cue-giver, provided the pointing gesture and E3 gave indications about signals to the cue-giver in a pseudo-random way (i.e., pointing at the same bowl was not allowed more than twice consecutively). The test procedure consisted of three phases. In a pre-trial motivation phase, to assess the subject’s interest in food, E2 positioned two stacked bowls 40 cm in front of him and at about 250 cm away from the dog, putting a piece of food (i.e., sausage) in the bowls once the dog had paid attention to him. Then, E1 released the dog to allow him to eat the food. After four trials, the bowls were then inverted to evenly distribute the smell of food within the other bowl for the following four trials. During the following test phase, E2 moved the bowls laterally 2 m apart, and if necessary, called for the dog’s visual attention. Afterward, E2 provided dynamic proximal pointing: kneeling and looking away from the dog, E2 extended the arm laterally pointing to one of the bowls with the finger and remaining motionless until the end of the trial. The distance between E2’s finger and the edge of the bowl was about 10–15 cm. As soon as E2 indicated the bowl, E1 released the dog. Every trial ended when the dog chose one of the bowls within 10 seconds, or after 10 seconds if the dog did not make a choice. The bowl was considered chosen when the dog approached it with the muzzle (i.e., the dog moved towards the target with the nose within 10 cm of the bowl). In this case, E2 dropped a piece of food inside the chosen bowl whereas no food was given to the dog for the incorrect choice. Every two test phase trials, an inter-motivation trial (identical to the pre-trial motivation phase), aimed at controlling the dog’s ongoing motivation and preventing any unwanted learning effect was performed. 

### 2.3. Data Analysis

For the data analysis, the frequency of correct, wrong and no-choices was collected. The latency of the correct choices (i.e., the time elapsed starting from the experimenter pointing signal until the dog made its choice) was also considered. Given that wrong choices were very limited in both groups (i.e., a maximum of two incorrect choices in about 10% of dogs in each of the two groups), the frequency and the latency of incorrect choices were not considered in the analysis because of the low statistical power. A correlation between the latency of the correct choices and the frequency of correct responses was tested by the Spearman’s rho. All data were obtained from the analysis of videos and collected using the Solomon Coder beta^®^ 14.05.19 (ELTE TTK, Budapest, Hungary). The reliability test was assessed by comparing data collected from 20% videos by another coder. The minimal agreement between coders in the measurements was 90%. After verifying that the data were not normally distributed by using the Kolmogorov–Smirnov test, the Mann-Whitney U test was applied to compare the frequencies of the correct choices and no-choices and the latencies of the correct ones between IF and GF. All statistical analyses were performed with IBM SPSS Statistics 24 software (IBM corporation, NY, USA). The experimental study was approved by the Ethical Animal Care and Use Committee of the University of Naples Federico II (protocol number 2017/0025509).

## 3. Results and Discussion

**Figure 1:**
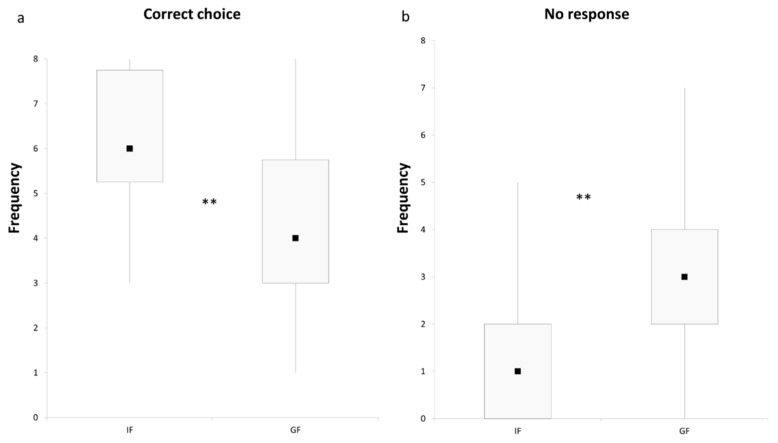
The frequency of correct choices (**a**) and no-choices (**b**) of intact (IF) and gonadectomized (GF) female dogs. Black squares: medians; boxes: quartiles; thin vertical lines: minimum and maximum values. ** *p* < 0.01.

The number of correct responses differs significantly between IF and GF groups, with the IF following the pointing gestures more often than the GF (Mann-Whitney U test: U = 99.5, *p* < 0.01; Figure 1a). A significantly higher no-choice frequency was recorded for the GF group compared to the IF group (Mann-Whitney U test: U = 101, *p* < 0.01; Figure 1b). 

**Figure 2:**
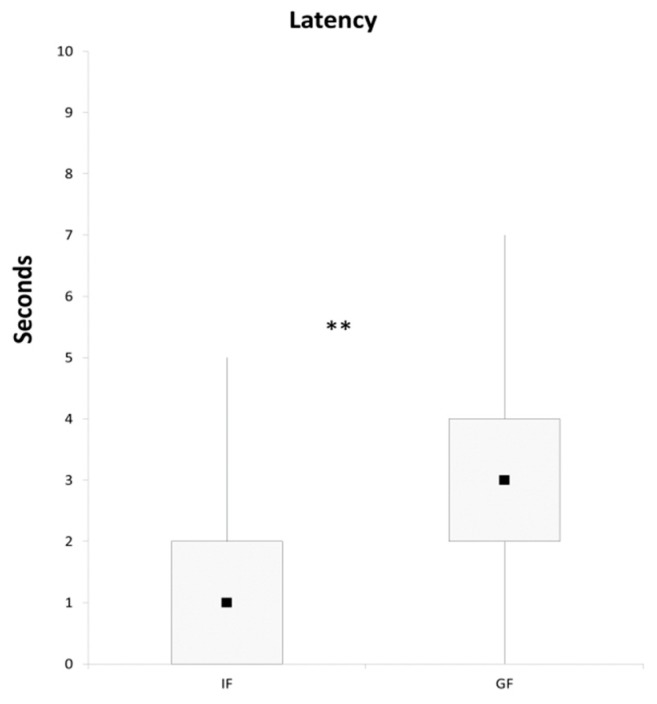
The latency of correct choices of intact (IF) and gonadectomized (GF) female dogs. Black squares: medians; boxes: quartiles; thin vertical lines: minimum and maximum values. ** *p* < 0.05.

The difference in the number of correct responses between the two groups could be explained by the better ability of the IFs to follow the pointing signal. Alternatively, the higher number of no-choices in the GF group could account for this result. The reasons for the absence of responses are debated in the literature and it is still unclear whether this depends on a dog’s lack of interest in the task or misinterpretation of the signals [9]. Irrespective of the group they belong to, all the dogs tested in this research showed a constant interest in food, and never failed any inter-motivation trial. Thus, rather than the GF group being less motivated to participate in the test, greater difficulty in reading the pointing gesture in this group might better explain the results. The IF group also showed lower latency in responding correctly than the GF (Mann-Whitney U test: U = 97.5, *p* < 0.01; Figure 2) and the latency of the correct choices was negatively correlated with the frequency of correct responses (Spearman correlation: r_s_ = −0.79, *p* < 0.01). 

Our results agree with a study on border collies in which speed and accuracy were positively correlated when solving a point following test [14]. Previous studies have demonstrated that ovariectomy influences rat locomotor activity in different tasks [15]. Moreover, a correlation between the reaction time in voluntary responses and the levels of female hormones was also demonstrated [16]. Reaction time has also been considered as an index of neuronal processing speed [17] and it is used to assess the ability to concentrate on a specific stimulus and respond accordingly [18]. It is not easy to find a causal effect between ovarian hormones and our results. Sex hormones were not quantified in our experimental subjects, thus whether the levels of circulating hormones were different in our samples is unknown. However, although in some cases oestradiol changes after ovariectomy are not reported in anoestrus [19], it has been demonstrated that ovariectomy in dogs reduces the levels of female sex hormones [19,20]. If so, it is possible that the lower female sex hormone levels in the GF group could be responsible for the inferior performances in both locomotor activity and responses. It must be mentioned that ovarian steroidogenesis has been reported in many other tissues such as the brain, in mammals [21] to invertebrate [22], and that brain could adapt the levels of neurosteroids after ovariectomy [23]. Indeed, hippocampal oestradiol levels in ovariectomized female rats remain unchanged [24]. However, in our study on dogs such compensatory adaptation (if any) was not enough to prevent an adverse effect on social cognition. It should be emphasized that ovarian hormones might not enhance learning in the same way during the acquisition phase of a task and in the working memory when the task needs to be repeated [25]. Although we were able to note difficulties in complying with the pointing gesture during the test, our present data does not allow us to disentangle whether the poorer performances in ovariectomized females are due to an impaired learning process in the acquisition phase of the pointing gesture in human families. Another limitation of our research is that only two breeds were studied, and thus, other breeds should be studied before making a generalization.

## 4. Conclusions

In conclusion, our results provide further evidence of the detrimental effect of ovariectomy on dogs’ socio-cognitive skills. The negative effects of ovariectomy in social cognition shown by the present results match similar outcomes in spatial cognition [3]. This is not surprising considering that a positive correlation exists between spatial cognition (i.e., four versions of a detour test) and social cognition (i.e., following human pointing) performances [14]. Further investigations are necessary to clarify which ovarian hormone could be involved in the regulation of the behavioral outcomes in dogs. We believe that our results, which report clear evidence of impaired communicative skills in ovariectomized dogs, can stimulate future research. It would be extremely interesting to broaden this kind of research to dogs of different ages, to assess if socio-cognitive impairment in gonadectomised dogs is e age-dependent, considering that the ideal age for the elective surgery (if necessary) does not exist [26].
